# Boeravinone A Alleviates Oxidative Stress and Inflammation in LPS-Induced Acute Kidney Injury by Targeting PGK1

**DOI:** 10.3390/antiox15070900

**Published:** 2026-07-20

**Authors:** Yi Lan, Lunqiong Ai, Liqing Tang, Nan Wang, Honghong Zhan, Han Yuan, Min Chen

**Affiliations:** Chongqing Key Laboratory of New Drug Screening from Traditional Chinese Medicine, College of Pharmaceutical Sciences, Southwest University, Chongqing 400715, China; lihuazou@email.swu.edu.cn (Y.L.); qwe28256@email.swu.edu.cn (L.A.); tlqlily@email.swu.edu.cn (L.T.); w112023329386383@swu.edu.cn (N.W.); zhh970924@email.swu.edu.cn (H.Z.); yh1943@swu.edu.cn (H.Y.)

**Keywords:** *Oxybaphus himalaicus* Edgew., Boeravinone A, acute kidney injury, Phosphoglycerate kinase 1, oxidative stress, inflammation

## Abstract

*Oxybaphus himalaicus* Edgew. is a traditional Tibetan medicinal plant used to treat nephritis and edema; however, its active constituents and the molecular mechanisms underlying its renoprotective properties remain poorly elucidated. This study investigated the pharmacological activity and mechanism of Boeravinone A (BA), a major constituent of *O. himalaicus*, in lipopolysaccharide (LPS)-induced acute kidney injury (AKI). A mouse model of LPS-induced AKI and LPS-stimulated RAW264.7 macrophages were used to evaluate the anti-inflammatory and renoprotective effects of BA in vivo and in vitro. Activity-based protein profiling (ABPP) was performed to identify potential molecular targets, followed by validation using isothermal titration calorimetry (ITC), cellular thermal shift assay (CETSA), and drug affinity responsive target stability (DARTS) assays. The functional role of the identified target was further examined using shRNA-mediated knockdown and virtual knockout analysis. BA dose-dependently attenuate LPS-induced renal injury and reduced inflammatory responses. Phosphoglycerate kinase 1 (PGK1) was identified as a direct target of BA. Mechanistically, BA activated the Kelch-like ECH-associated protein 1–nuclear factor erythroid 2-related factor 2 (Keap1–Nrf2) pathway through PGK1, enhanced the expression of antioxidant enzymes such as *Nqo1*, and reduced the production of pro-inflammatory cytokines, including IL-1β and IL-6. Virtual knockout of PGK1 in macrophages further supported its regulatory role in this pathway. These findings suggest that BA exerts renoprotective effects by targeting PGK1 and activating the Keap1-Nrf2 pathway, thereby reducing oxidative stress and inflammation. This study provides a pharmacological basis for the traditional use of *O. himalaicus* and supports BA as a potential candidate for mechanism-based intervention in AKI.

## 1. Introduction

Acute kidney injury (AKI) is a clinically important condition characterized by a rapid decline in renal function and is associated with high morbidity and mortality. Current treatment for AKI mainly relies on supportive interventions, including fluid management, renal replacement therapy (such as dialysis), and treatment of the underlying cause [1]. However, these therapeutic strategies do not directly target the molecular mechanisms driving renal injury. In addition, the therapeutic efficacy remains limited and insufficient to prevent persistent renal dysfunction or progression to chronic kidney disease [2,3]. Therefore, the development of mechanism-based treatment approaches for AKI remains an important research and clinical priority.

The pathogenesis of AKI is complex and involves multiple interconnected processes, among which inflammation and oxidative stress play central roles [4,5]. In LPS-induced AKI, activation of the innate immune response promotes nuclear factor-kappa B (NF-κB) signaling and increases the production of pro-inflammatory cytokines, including tumor necrosis factor-α (TNF-α) and interleukin-1β (IL-1β) [6,7]. These inflammatory mediators contribute to excessive production of reactive oxygen species (ROS), disruption of mitochondrial function, and further aggravation of oxidative stress. ROS can also act as signaling molecules that sustain inflammatory pathways, including NF-κB signaling, thereby creating a positive feedback loop between oxidative stress and inflammation during AKI progression [8,9].

The Keap1-Nrf2 pathway is a key antioxidant defense system that protects cells against oxidative damage [10]. Under basal conditions, Nrf2 is bound by Keap1, leading to its ubiquitination and proteasomal degradation. However, Nrf2 dissociates from Keap1 and translocates into the nucleus, where it binds to antioxidant response elements and promotes the transcription of antioxidant and cytoprotective genes [11,12]. Activation of Nrf2 has been shown to alleviate oxidative injury and inflammatory responses in experimental models of AKI, whereas Nrf2 deficiency exacerbates renal dysfunction and histological damage [13,14]. Thus, regulation of the Keap1-Nrf2 pathway represents a promising strategy for reducing oxidative stress-mediated renal injury.

PGK1 is a glycolytic that catalyzes the reversible conversion of 1,3-bisphosphoglycerate (1,3-BPG) to 3-phosphoglycerate (3-PG) and plays an essential role in cellular energy metabolism [15]. In addition to its established metabolic function, PGK1 has been associated with the regulation of oxidative stress, inflammation, and cellular stress responses [16,17]. PGK1 has long been reckoned as a negative regulator of Nrf2 signaling. Both genetic depletion and chemical inhibition of PGK1 activate Nrf2 signaling. However, no well-recognized evidence explains the molecular basis of PGK1 inactivating Nrf2 signaling. A previous study showed that PGK1 inhibition or depletion will lead to Keap1 methylglyoxal modification, thereby activating the Nrf2 signaling cascade [18,19]. These findings indicate that PGK1 may serve as a potential regulatory node linking metabolic adaptation, antioxidant defense, and inflammatory regulation in AKI.

*Oxybaphus himalaicus* Edgew. is a traditional Tibetan medicinal plant belonging to the group known as the “five roots” in Tibetan medicine. It has traditionally been used for conditions related to nephritis, edema, lower-limb cold syndrome, and yellow water disease and is believed to support kidney function and strengthen the body. Our previous study showed that the total extract of *O. himalaicus* improved LPS-induced AKI [20]. Boeravinone A (BA), one of the major constituents of *O. himalaicus*, has been reported to possess anti-inflammatory activity [21]. However, whether BA contributes to the renoprotective effects of *O. himalaicus* and the molecular mechanisms underlying its activity remain unclear. In this study, we investigated the renoprotective effects of BA and explored its underlying mechanism using a mouse model of LPS-induced AKI and LPS-stimulated RAW264.7 macrophages. Activity-based protein profiling was used to identify potential molecular targets of BA, followed by target validation and mechanistic analysis. We further examined whether BA regulates oxidative stress and inflammation through PGK1-mediated activation of the Keap1-Nrf2 pathway. This study aimed to provide mechanistic evidence for the renoprotective activity of BA and a pharmacological basis for the traditional use of *O. himalaicus* in kidney-related disorders.

## 2. Materials and Methods

### 2.1. Reagents

BA was isolated from *O. himalaicus* with a purity of 99%, elucidated by 1H-NMR and 13C-NMR (Figure S1). LPS was purchased from Beyotime (Shanghai, China). Anti-TNF-α (R1203-1) and anti-IL-1β (ET1701-39) antibodies were obtained from HUABIO (Hangzhou, China). Anti-β-actin (66009-1-Ig) and anti-IL-6 (26404-1-AP) were purchased from Proteintech (Wuhan, China). Anti-Keap1 (10503-1-AP) and Nrf2 (AF7623) antibodies, PCNA(AF0261) antibodies, and HRP-conjugated goat anti-rabbit IgG (A0208) and goat anti-mouse IgG (A0216) were purchased from Beyotime (Shanghai, China). Anti-PGK1 (A12686) antibodies were obtained from ABclonal (Wuhan, China).

### 2.2. Animals

Male C57BL/6J mice aged 6 to 8 weeks and weighing 20 ± 2 g were purchased from Hunan SJA Laboratory Animal Co., Ltd. The mice were housed under specific pathogen-free (SPF) conditions at 25 °C and 50% relative humidity with free access to food and water. All animal procedures were approved by the Animal Ethics Committee of Southwest University (IACUC-20240520-06).

After one week of acclimatization, the mice were randomly divided into five groups: control group (*n* = 8), AKI model group (*n* = 8), BA low-dose group (10 mg/kg, *n* = 8), BA high-dose group (20 mg/kg, *n* = 8), and dexamethasone group (5 mg/kg, *n* = 8). The AKI model was established by intraperitoneal injection of LPS at a dose of 10 mg/kg. Mice in the control group received an equivalent volume of sterile PBS. After injection, mice were fasted but allowed free access to water. At 12 h after LPS administration, mice were anesthetized with isoflurane and sacrificed. Orbital blood and kidney tissues were collected for subsequent analyses. In all subsequent tissue-based experiments, three kidney samples were randomly selected from each group, which consisted of eight mice.

### 2.3. Serological and Histopathological Examination

Blood samples were centrifuged at 8000 rpm for 10 min to obtain serum. Serum blood urea nitrogen (BUN) and serum creatinine (Cre) levels were measured using commercial kits (Nanjing Jiancheng Bioengineering Institute, Nanjing, China) according to the manufacturer’s instructions. For histopathological analysis, kidney tissues were fixed in 4% paraformaldehyde, dehydrated, embedded in paraffin, and sectioned at a thickness of 4 μm. The sections were then subjected to hematoxylin–eosin (H&E) and immunohistochemistry (IHC).

### 2.4. Cell Culture

RAW264.7 macrophages and HEK293T cells were obtained from the Cell Bank of the Chinese Academy of Sciences. RAW264.7 macrophages were cultured in RPMI-1640 medium, whereas HEK293T cells were maintained in DMEM/F12 medium (BasalMedia, Shanghai, China). Both media were supplemented with 10% FBS (BDBIO, Hangzhou, China) and 100 U/mL penicillin-streptomycin (Beyotime, Shanghai, China). All cells were incubated at 37 °C in a humidified incubator containing 5% CO_2_.

### 2.5. Immunofluorescence Staining

RAW264.7 macrophages were seeded into 12-well plates containing coverslips and cultured for 24 h. The cells were then treated with 20 μM of BA and 1 μg/mL of LPS for an additional 24 h. The concentrations of BA used in the present study were selected based on our previous optimization experiments. Under these experimental conditions, BA did not exhibit detectable cytotoxicity in the investigated cell line. Immunofluorescence staining was performed as previously described [20]. Images were captured using an inverted microscope. Immunohistochemical images were quantified using ImageJ 1.54g software (National Institutes of Health, Bethesda, MD, USA). Freehand ROI tools were used to delineate only renal tubular epithelial regions (glomeruli and large blood vessels were excluded). The “%Area” (positive area fraction) and AOD (average optical density) of each ROI were calculated. Six random non-overlapping cortical fields per section were captured under 200× magnification, and the average value of six visual fields was used for statistical analysis. All image acquisition and quantification were performed by two blind investigators.

### 2.6. Cytoplasmic and Nuclear Protein Extraction

Cells were seeded and treated as described above. After 24 h, cells were harvested and washed with ice-cold PBS. For cytoplasmic protein extraction, cell pellets were lysed in 300 μL of Buffer A containing 10 mM of HEPES pH 7.9, 10 mM of KCl, 1 mM of EGTA, 1 mM of DTT, 0.5% NP-40, and 1 mM of PMSF for 15 min on ice. The lysates were then centrifuged at 12,000× *g* for 5 min at 4 °C, and the supernatant was collected as the cytoplasmic fractions. The remaining pellets were lysed with 100 μL of nuclear lysis buffer consisting of RIPA supplemented with 1% PMSF and super nuclease for 40 min on ice. After centrifugation, the supernatants were collected as the nuclear fractions. Protein concentrations were determined using the BCA assay. The samples were mixed with loading buffer and boiled for 10 min before further analysis.

### 2.7. ROS Detection

RAW264.7 macrophages were seeded into 6-well plates and cultured for 24 h. Cells were then treated with 20 μM of BA and 1 μg/mL of LPS for an additional 24 h. Intracellular ROS levels were detected using an ROS assay kit (Beyotime, Shanghai, China) according to the manufacturer’s instructions. Images were captured using an inverted microscope.

### 2.8. Western Blotting

RAW264.7 macrophages were seeded in 6-well plates at a density of 5 × 10^5^ cells per well. After the cells adhered for 24 h, they were co-incubated with different concentrations of BA or the control solvent (DMSO) and LPS (1 μg/mL) for 24 h. Proteins were extracted from kidney tissues and RAW264.7 macrophages using RIPA lysis buffer (Dingguochangsheng Biotechnology, Beijing, China) supplemented with protease inhibitor cocktail (Servicebio, Wuhan, China). Protein samples were separated by SDS-PAGE and transferred onto polyvinylidene difluoride (PVDF) membranes. The membranes were blocked with 5% skim milk at room temperature for 2 h, followed by overnight incubation with primary antibodies at 4 °C. After washing, the membranes were incubated with HRP-conjugated secondary antibodies at room temperature for 2 h. Protein bands were visualized using a chemiluminescence reagent (Proteintech, Wuhan, China) and analyzed using ImageJ 1.54g software (National Institutes of Health, Bethesda, MD, USA).

### 2.9. Quantitative Real-Time Polymerase Chain Reaction (qRT-PCR)

RAW264.7 macrophages were seeded in 6-well plates at a density of 5 × 10^5^ cells per well. After the cells adhered for 24 h, they were co-incubated with different concentrations of BA or the control solvent (DMSO) and LPS (1 μg/mL) for 6 h. Total RNA was extracted from RAW264.7 macrophages using the Quick RNA extraction kit (Accurate Biotechnology, Changsha, China). RNA was reverse-transcribed into cDNA using the Evo M-MLV reverse transcription kit with gDNA remover (Accurate Biotechnology, Changsha, China). Quantitative real-time PCR was performed using the SYBR Green premix Pro-Taq HS qPCR kit (Accurate Biotechnology, Changsha, China). mRNA expression levels were normalized to *Actb*. Relative expression levels were calculated using the 2^−ΔΔCt^ method, where ΔCt = Ct_target − Ct_*Actb* and ΔΔCt = ΔCt_treated − ΔCt_control (average). Primer sequences and information are listed in Table S1 in the Supplementary Materials.

### 2.10. Drug Affinity Responsive Target Stability Assay (DARTS)

RAW264.7 macrophages cultured in 10 cm dishes were lysed with RIPA lysis buffer supplemented with protease inhibitor cocktail. After centrifugation with 12,000 rpm at 4 °C, the supernatants were collected and divided into two aliquots. The aliquots were incubated with dimethyl sulfoxide (DMSO) or 20 μM of BA at room temperature for 50 min. Each aliquot was then subdivided into eight aliquots and incubated with Pronase E (Solaribio, Beijing, China) at protein-to-protease ratios ranging from 1:1600 to 1:100. The mixtures were incubated at 37 °C for 15 min and then centrifuged at 5000 rpm for 5 min at room temperature. The supernatants were collected for Western blotting analysis.

### 2.11. Cellular Thermal Shift Assay (CETSA)

RAW264.7 macrophages cultured in 10 cm dishes were lysed with RIPA lysis buffer supplemented with a protease inhibitor cocktail. After centrifugation at 12,000 rpm at 4 °C, the supernatants were collected and divided into two aliquots. The aliquots were incubated with DMSO or 20 μM of BA at room temperature for 50 min. Each aliquot was then subdivided into six aliquots and heated at gradient temperatures ranging from 48 °C to 78 °C for 10 min. The samples were centrifuged at 5000 rpm for 5 min at room temperature, and the supernatants were collected for Western blotting analysis.

### 2.12. Recombinant Protein Expression and Purification

The pET28a-PGK1 plasmid was transformed into *Escherichia coli* BL21(DE3) competent cells, and single colonies were selected for amplification. When the culture reached an OD600 of 0.8, 0.5 mM of isopropyl β-D-thiogalactopyranoside (IPTG) was added to induce protein expression, and the culture was incubated at 16 °C for 15 h. The bacteria were harvested and lysed by sonication in 50 mL of lysis buffer containing PBS, 0.1% Tween 20, 0.1% β-mercaptoethanol, and 1 mM of PMSF. The lysate was centrifuged at 12,000 rpm for 30 min at 4 °C, and the supernatant was collected. PGK1 was purified using His-tag purification resin (Beyotime, Shanghai, China). The eluted protein was desalted by dialysis and concentrated by ultrafiltration. The purified protein was verified by SDS-PAGE.

### 2.13. Isothermal Titration Calorimetry (ITC) Analysis

ITC experiments were performed at 25 °C using a MicroCal ITC200 microcalorimeter (Northampton, MA, USA). PGK1 was thoroughly dialyzed in PBS, and the BA stock solution was diluted using the same dialysis buffer. DMSO was added to the protein solution at the same final concentration as that in the BA solution. PGK1 protein (20 μM) was loaded into the sample cell, and BA (400 μM) was loaded into the injection syringe. A reference titration was performed by titrating BA into the buffer to correct for dilution heat. The stirring speed was set to 1000 rpm. Thermodynamic parameters were analyzed using MicroCal Origin 7.0 software (Northampton, MA, USA).

### 2.14. Molecular Docking

The crystal structure of human PGK1 was downloaded from the RCSB PDB database (PDB: 9KER). Molecular docking was performed using Sybyl-X 2.0 software (Tripos Associates, St. Petersburg, FL, USA). The potential binding pocket in PGK1 was identified by multi-channel surface scanning. The docking complex with the highest docking score was selected for visualization using PyMOL 3.7 software (Schrödinger, LLC, New York, NY, USA).

### 2.15. Bioinformatics Analysis

Single-cell RNA-seq (scRNA-seq) data from LPS-stimulated RAW264.7 macrophages were downloaded from the Gene Expression Omnibus (GEO) database under accession number GSE207902, sample GSM6322830. The VEH_LPS 1 h condition was used for analysis. All analyses were performed in R version 4.2. The code was generated by AI.

Raw count matrices were processed using the Seurat package version 5.1 [22]. Highly variable genes (HVGs) were identified using the variance-stabilizing transformation (VST) method, and the top 2000 genes were selected. Genes with zero total counts across all cells were excluded. The final gene set was used for the virtual knockout (KO) analysis.

Virtual KO analysis of PGK1 was performed using the R package scTenifoldKnk version 1.2 [23]. Briefly, scTenifoldKnk constructs cell-specific gene regulatory networks through tensor decomposition of subsampled count matrices and simulates in silico deletion of the target gene by removing its network edges. The perturbed network is then compared with the unperturbed state to estimate gene-level regulatory effects. The following parameters were used: number of subnetworks, nc_nNet = 15; cells per subnetwork, nc_nCells = 500; and parallel cores = 8. A random seed of 42 was set prior to analysis to ensure reproducibility. Gene-level perturbation effects were quantified using z-scores, and genes with a Benjamini–Hochberg-adjusted *p*-value < 0.05 were considered significantly affected. The target gene, PGK1, was excluded from downstream reporting.

Gene Ontology (GO) Biological Process (BP) enrichment analysis of significantly affected genes was performed using the clusterProfiler package (v4.1) [24], with the full set of analyzed genes used as the statistical background. Enrichment significance was determined using an adjusted *p* < 0.05 after Benjamini-Hochberg correction. Mouse gene symbols were converted to Entrez IDs using the org.Mm.eg.db annotation package version 3.17. The complete analysis code, including all R scripts and a step-by-step pipeline, is publicly available at https://sandbox.zenodo.org/records/514681 (accessed on 17 June 2026).

### 2.16. Construction of shRNA

To establish RAW264.7 cells with stable Pgk1 knockdown, lentiviral particles were first packaged in HEK293T cells. Briefly, HEK293T cells were cultured to 70–80% confluence and co-transfected with pMD2.G, psPAX2, and the pLKO.1-shRNA plasmid targeting mouse PGK1. At 8 h after transfection, the medium was replaced with complete medium containing 10% FBS. After 48 h, the culture supernatant was collected, filtered through a 0.22 μm micropore filtering membrane, aliquoted, and stored at −80 °C. RAW264.7 cells at approximately 60% confluence were then infected with the lentiviral supernatant containing 8 μg/mL of polybrene. After 24 h, the medium was replaced with fresh medium. At 48 h after infection, puromycin was added at a final concentration of 4 μg/mL, and the cells were selected continuously for 7–10 days. Puromycin-containing medium was replaced every 1–2 days. After all uninfected control cells had died, *Pgk1* knockdown efficiency was verified by Western blotting. The shRNA sequences are provided in Table S2.

### 2.17. Activity-Based Protein Profiling (ABPP)

RAW264.7 macrophages were lysed in RIPA buffer to obtain total cellular proteins. The protein lysate was equally divided into two aliquots. One aliquot was incubated with 20 μM of BP, whereas the other was treated with a mixture of 20 μM of BP and 100 μM of BA to serve as competitive binding control. Both reactions were performed at 4 °C for 3 h.

Following incubation, a click chemistry reaction was carried out by adding a reaction mixture containing 100 mM of Biotin-N3, 100 mM of TBTA, 0.5 M of TCEP, and 1 M of CuSO_4_, followed by gentle rotation in the dark for 2 h. Proteins were subsequently precipitated by adding four volumes of pre-cooled acetone and incubating the samples at −20 °C overnight.

The resulting protein pellets were resuspended in 0.5% SDS/PBS with sonication. The SDS concentration was then reduced to 0.2% by dilution with PBS before incubation with streptavidin-coated magnetic beads at room temperature for 1 h to enrich biotin-labeled proteins. After extensive washing with 0.5% SDS/PBS five times to remove nonspecific binding, the captured proteins were eluted by boiling the beads in SDS loading buffer. The eluates were separated by SDS-PAGE and subjected to LC-MS/MS analysis.

### 2.18. Statistical Analysis

Statistical analysis was performed using GraphPad Prism 10.0 software (Boston, MA, USA). Data are presented as mean ± standard deviation (SD). Statistical differences among multiple groups were analyzed using one-way analysis of variance (ANOVA) followed by Tukey’s multiple comparison test. Comparisons between two groups were performed using an unpaired Student’s *t*-test. A *p*-value < 0.05 was considered statistically significant. All the parametric tests conducted in our study underwent Shapiro-Wilk tests to assess the normality and equal variance assumptions. Only datasets that met these assumptions were used for significant analysis.

For virtual KO analysis of PGK1, gene-level perturbation effects were quantified using z-scores, and significantly affected genes were defined by an Benjamini-Hochberg-adjusted *p* < 0.05. For GO BP enrichment analysis, the statistical significance was determined using the hypergeometric test with false discovery rate (FDR) correction, and terms with adjusted *p* < 0.05 were considered significantly enriched.

## 3. Results

### 3.1. BA Alleviates Renal Pathological Damage and Inflammatory Responses in Mice with LPS-Induced AKI

**Figure 1:**
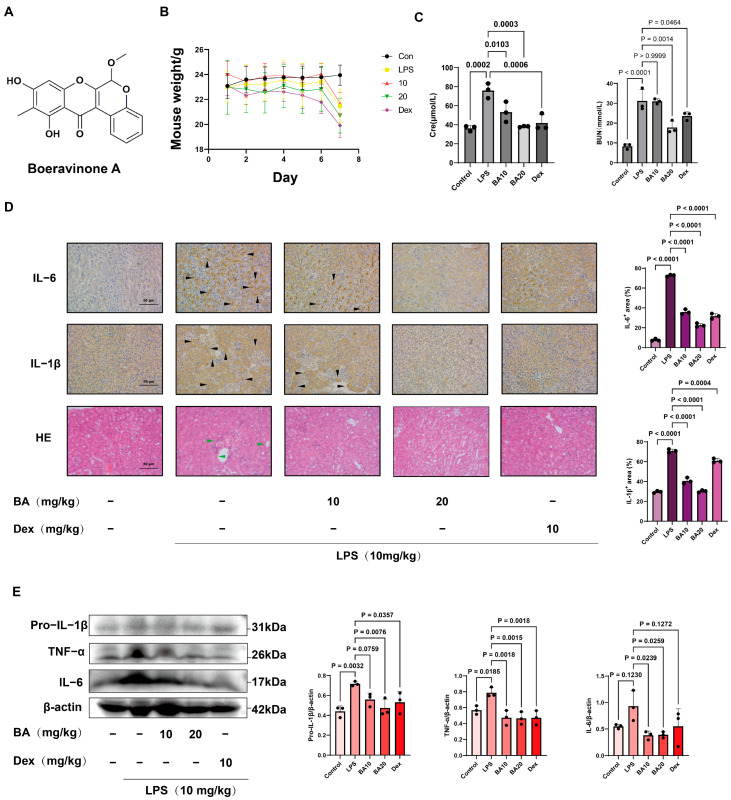
BA alleviates renal pathological damage and inflammatory responses in mice with LPS-induced AKI. (**A**) Chemical structure of BA. (**B**) Changes in mouse body weight during the experiment (*n* = 8). (**C**) Serum Cre and BUN levels (*n* = 3). (**D**) Representative images of renal IL-6 and IL-1β IHC staining (Brown staining indicates positive immunoreactivity, and black arrowheads indicate representative positive staining) and H&E staining (Green arrowheads indicate representative histopathological lesions), with corresponding quantification of IHC-positive areas. Scale bar: 50 μm (*n* = 3). (**E**) Western blot analysis of pro-IL-1β, TNF-α, and IL-6 protein expression in renal tissues, with β-actin used as the internal control (*n* = 3). Data is presented as mean ± SD.

Continuous monitoring of physiological parameters showed no significant differences in body weight among mice treated with LPS or those receiving the BA intervention, suggesting that BA did not induce apparent acute systemic toxicity at the tested dosage (Figure 1B). Biochemical analysis showed that LPS markedly increased serum Cre and BUN levels, whereas BA treatment reduced these elevations in AKI mice (Figure 1C). H&E staining further revealed severe renal pathological injury in the LPS group, characterized by extensive vacuolar swelling, necrosis, and sloughing of renal tubular epithelial cells, accompanied by massive inflammatory cell infiltration. In contrast, BA treatment reduced tubular necrosis and inflammatory infiltration, and the renal parenchymal structure was relatively preserved. Consistently, IHC staining showed that LPS increased the expression of pro-inflammatory proteins IL-1β and IL-6 in the tubulointerstitial area, whereas BA treatment markedly decreased their expression in the renal tubular tissues (Figure 1D). Western blotting further confirmed that BA reduced the protein levels of IL-1β, TNF-α, and IL-6 in kidney tissues compared with those in the LPS group (Figure 1E). These results indicate that BA attenuates LPS-induced renal injury and suppresses inflammatory responses in vivo.

### 3.2. PGK1 Is Identified as a Potential Molecular Target of BA

**Figure 2:**
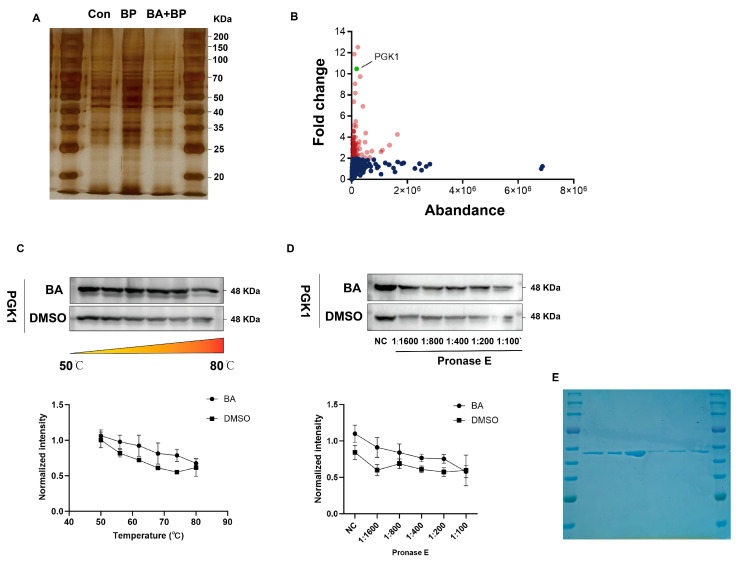

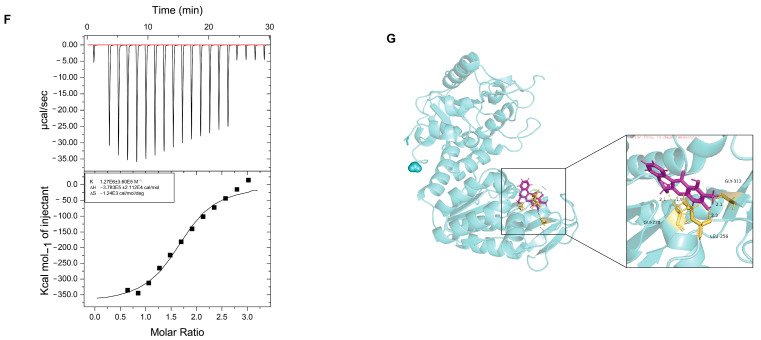
PGK1 is identified as a potential molecular target of BA. (**A**) Silver-stained SDS-PAGE gel showing proteins pulled down by BA. (**B**) Volcano plot of proteins identified by mass spectrometry, with PGK1 highlighted as the candidate target. (**C**) CETSA showing the thermal stability of PGK1 in BA- and DMSO-treated cells (*n* = 3). (**D**) DARTS assay showing the effect of BA on PGK1 stability against Pronase E digestion (*n* = 3). Assay demonstrating the effect of BA on PGK1 protein stability against Pronase E digestion (*n* = 3). (**E**) Coomassie Blue staining of purified PGK1 protein. (**F**) ITC analysis of the binding affinity between BA and PGK1. (**G**) Molecular docking model showing the predicted binding mode of BA with PGK1. Data are presented as the mean ± SD. *n* = 3 for CETSA and DARTS analyses.

ABPP was performed in LPS-stimulated RAW264.7 macrophages to identify potential molecular targets of BA. Among the identified candidate target proteins, PGK1 was selected for further validation based on its potential relevance to inflammation and oxidative stress regulation (Figure 2A,B). To determine whether BA directly interacts with PGK1, CETSA and DARTS assays were performed. CETSA showed that BA increased the thermal stability of PGK1 compared with DMSO treatment (Figure 2C). Consistently, DARTS showed that BA enhanced the resistance of PGK1 to Pronase E-mediated proteolytic digestion (Figure 2D). These results suggest direct interaction between BA and PGK1 in cells. To further validate this interaction, recombinant human PGK1 was purified (Figure 2E) and subjected to ITC analysis. The binding affinity between BA and PGK1 was confirmed, with a dissociation constant (*K_D_*) value of 0.787 μM (Figure 2F). Molecular docking analysis further predicted that BA could bind within the active pocket of PGK1, interacting with amino acid residues including LEU 256, GLY 238, and GLY 312 (Figure 2G). The docking score of 6.06 suggested a favorable binding interaction between BA and PGK1. These results support PGK1 as a potential molecular target of BA.

### 3.3. BA Activates the Keap1-Nrf2 Pathway and Enhances Antioxidant Responses

**Figure 3:**
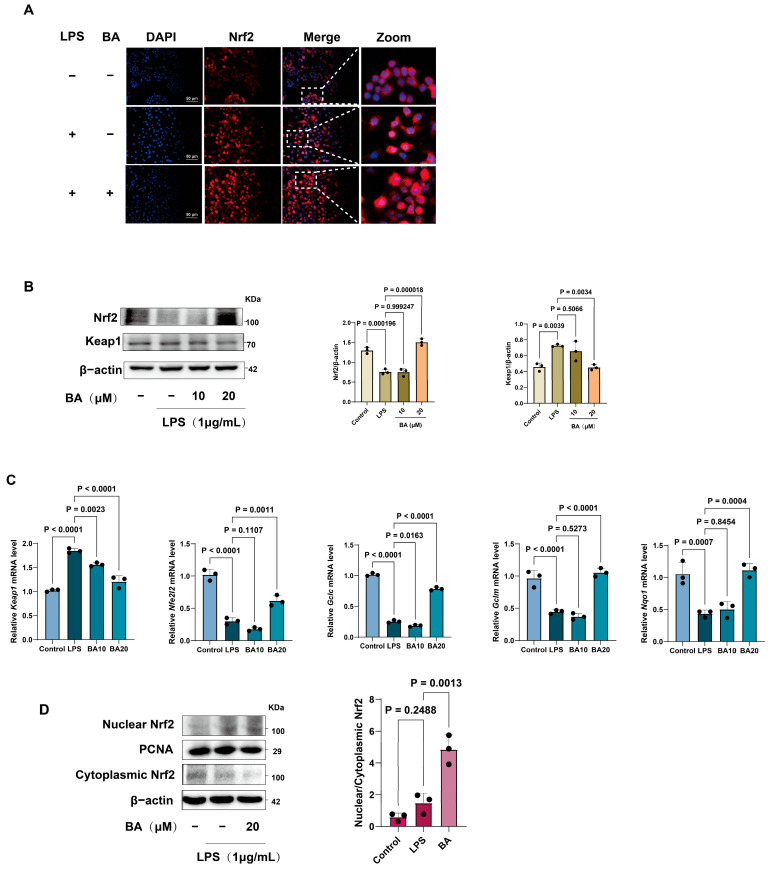
BA activates the Keap1-Nrf2 pathway in LPS-stimulated RAW264.7 macrophages. (**A**) Cells were co-incubated with LPS (1 μg/mL) and indicated concentrations of BA (20 μM) for 24 h, with immunofluorescence staining of Nrf2 (red) in control, LPS-treated, and BA-treated RAW264.7 macrophages. Nuclei were counterstained with DAPI (blue). Scale bar: 20 μm. (**B**) Western blot analysis of Keap1 and Nrf2 protein expression (*n* = 3). (**C**) Relative mRNA expression of *Nfe2l2*, *Keap1*, *Nqo1*, *Gclc*, and *Gclm*. (**D**) The nuclear and cytoplasmic Nrf2 protein levels were detected by Western blotting. (*n* = 3). Data are presented as the mean ± SD from three independent experiments.

We next investigated the regulatory effects of BA on the Keap1–Nrf2 signaling pathway in LPS-stimulated RAW264.7 macrophages. Immunofluorescence staining showed that LPS stimulation reduced nuclear Nrf2 accumulation, whereas BA treatment promoted Nrf2 translocation into the nucleus (Figure 3A). Western blotting further showed that LPS increased Keap1 protein expression and decreased Nrf2 protein expression, whereas BA treatment reversed these changes (Figure 3B). Consistent with these findings, qRT-PCR showed that BA regulated the mRNA expression of Keap1-Nrf2 pathway-related genes (Figure 3C). The expression of downstream antioxidant genes regulated by Nrf2 was further assessed. LPS stimulation decreased the mRNA expression of *Nqo1*, *Gclc*, and *Gclm*, whereas BA treatment significantly restored their expression levels (Figure 3C). These findings suggest that BA enhances the transcriptional activation of antioxidant defense genes under LPS-induced inflammatory conditions.

To further confirm the effect of BA on Nrf2 activation, nuclear and cytoplasmic Nrf2 protein levels were examined using Western blotting. Compared with M0-type macrophages, LPS induced mild activation of Nrf2, whereas BA further enhanced Nrf2 nuclear accumulation. Notably, pretreatment with 20 μM of BA significantly enhanced nuclear Nrf2 accumulation in LPS-stimulated RAW264.7 macrophages, accompanied by a marked reduction in cytoplasmic Nrf2 levels (Figure 3D). These results indicate that BA promotes Nrf2 nuclear translocation and activates Nrf2-mediated antioxidant signaling in LPS-stimulated macrophages.

### 3.4. Virtual KO of PGK1 Reveals Its Regulatory Role in Macrophage Inflammatory Responses

**Figure 4:**
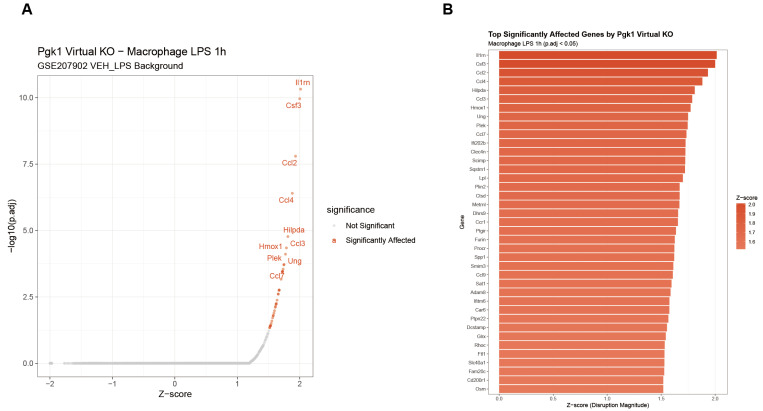

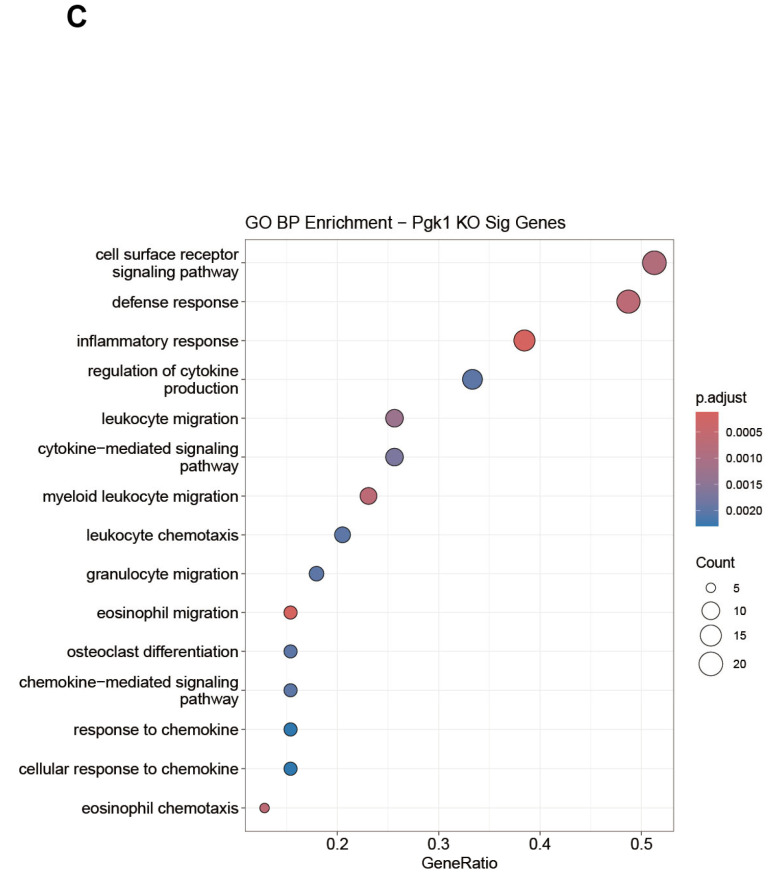
Virtual KO of PGK1 reveals its regulatory role in macrophage inflammatory responses. (**A**) Volcano plot showing significantly affected genes after PGK1 virtual KO in LPS-stimulated RAW264.7 macrophages. (**B**) Top genes significantly affected by PGK1 virtual KO, ranked by z-score. (**C**) GO BP enrichment analysis of genes affected by PGK1 virtual KO, showing enrichment in immune and inflammation-related biological processes.

ScTenifoldKnk was used to perform virtual KO analysis of PGK1 to further explore its potential regulatory function in LPS-stimulated RAW264.7 macrophages. Virtual KO of PGK1 markedly reshaped the gene regulatory profile and identified 38 significantly upregulated genes with an adjusted *p* < 0.05. Volcano plot analysis showed that the most significantly affected genes exhibited positive z-scores, indicating an overall transcriptional activation pattern after PGK1 perturbation (Figure 4A). Among the affected genes, *Il1rn*, *Hmox1*, and several chemokine family members, including *Ccl2* and *Ccl7*, showed prominent changes in expression (Figure 4B). These genes are closely associated with inflammatory regulation, immune cell recruitment, and stress responses, suggesting that PGK1 may participate in the regulation of macrophage inflammatory activity. GO BP enrichment analysis further showed that genes affected by PGK1 virtual KO were mainly enriched in biological processes related to immune system processes, defense responses, and responses to biostimulants (Figure 4C). These findings support the regulatory role of PGK1 in macrophage responses to LPS stimulation and are consistent with the experimental evidence linking PGK1 to inflammation-related signaling.

### 3.5. BA Targets PGK1 to Exert Antioxidant Effects

**Figure 5:**
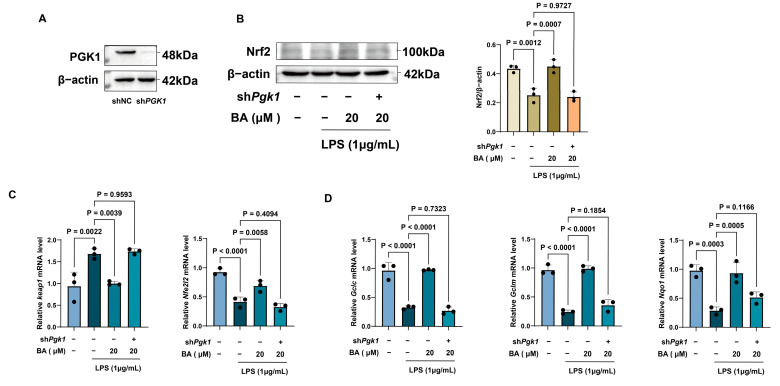

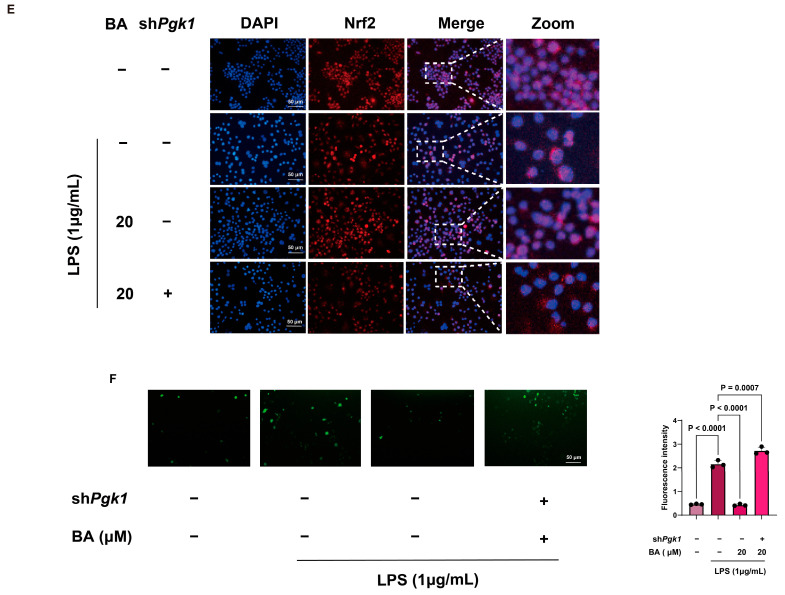
PGK1 is involved in BA-mediated activation of the Keap1-Nrf2 pathway and reduction in oxidative stress. (**A**) Western blot validation of *Pgk1* knockdown efficiency. Cells were treated with LPS (1 μg/mL) and BA (20 μM) for 24 h. (**B**) Western blot analysis of Nrf2 protein expression in LPS-stimulated shNC and sh *Pgk1* macrophages (*n* = 3). (**C**) qRT-PCR analysis of *Keap1* and *Nfe2l2* mRNA expression (*n* = 3). (**D**) qRT-PCR analysis of *Nqo1*, *Gclc*, *and Gclm* mRNA expression (*n* = 3). (**E**) Immunofluorescence staining showing Nrf2 nuclear translocation. Nrf2 is shown in red, nuclei are stained with DAPI (blue) (**F**) Representative ROS staining images in different treatment groups. ROS is shown in green (DCFH-DA) (*n* = 3). Data is presented as the mean ± SD.

To further determine whether the antioxidant effect of BA depends on PGK1, shRNA-mediated *Pgk1* knockdown was performed in RAW264.7 macrophages. Western blotting confirmed the knockdown efficiency of PGK1 (Figure 5A). In shNC macrophages, BA activated the Keap1-Nrf2 pathway under LPS-stimulated conditions, as indicated by changes in Nrf2 protein expression and Keap1-Nrf2 pathway-related gene expression. However, these effects were markedly weakened after *Pgk1* knockdown (Figure 5B,C). The effect of BA on downstream antioxidant genes was further examined by qRT-PCR. BA increased the mRNA expression of *Nqo1*, *Gclc*, and *Gclm* in LPS-stimulated shNC macrophages, whereas this regulatory effect was reduced in sh *Pgk1* macrophages (Figure 5D). Consistently, immunofluorescence staining showed that BA promoted Nrf2 nuclear translocation in shNC macrophages, but this effect was attenuated after *Pgk1* knockdown (Figure 5E). ROS production was then evaluated using DCFH-DA staining. LPS stimulation markedly increased intracellular ROS levels in RAW264.7 macrophages, whereas BA treatment reduced ROS accumulation. Notably, the ROS-lowering effect of BA was largely diminished after *Pgk1* knockdown (Figure 5F). Together, these results indicate that PGK1 is required for BA-mediated activation of the Keap1-Nrf2 pathway and suppression of oxidative stress in LPS-stimulated macrophages.

### 3.6. BA Activates the Keap1-Nrf2 Pathway in Kidney Tissues of Mice with LPS-Induced AKI

**Figure 6:**
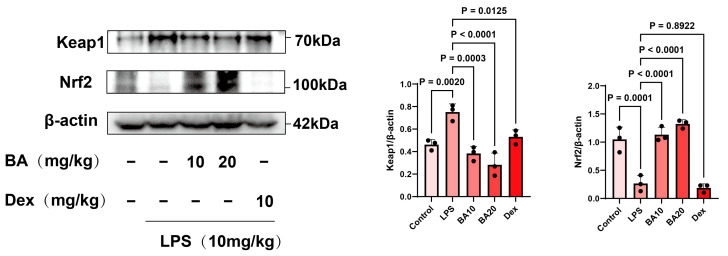
BA activates the Keap1-Nrf2 pathway in kidney tissues of mice with LPS-induced AKI. Western blot analysis of Keap1 and Nrf2 protein expression in kidney tissues from AKI mice. Data are presented as the mean ± SD (*n* = 3).

To further verify whether BA regulates the Keap1-Nrf2 pathway in vivo, Keap1 and Nrf2 protein expression levels were examined in kidney tissues from mice with LPS-induced AKI. Western blot analysis showed that LPS stimulation disrupted Keap1-Nrf2 pathway activation in kidney tissues, whereas BA treatment reversed these alterations (Figure 6). This result was consistent with the findings observed in RAW264.7 macrophages. These findings suggest that BA activates the Keap1-Nrf2 pathway both in vitro and in vivo, and BA treatment supports the attenuation of LPS-induced AKI through PGK1-associated activation of the Keap1-Nrf2 pathway.

## 4. Discussion

*O. himalaicus* is classified as one of the “Five Roots” in the Tibetan medical system. According to the Tibetan medical theory, *O. himalaicus* has traditionally been used to reinforce kidney function and strengthen the body [25]. The traditional indications of nephritis and edema are closely related to renal inflammation and fluid imbalance, which are also important pathological features of AKI in modern medicine. Previous studies have reported that extracts from *O. himalaicus* possess anti-inflammatory activity [21]. In the present study, we demonstrated that BA, the major constituent of *O. himalaicus,* attenuated renal dysfunction, pathological injury, and inflammatory responses in mice with LPS-induced AKI. Mechanistically, PGK1 was identified as a potential molecular target of BA. BA directly bound to PGK1 and activated the Keap1-Nrf2 pathway, thereby reducing oxidative stress and pro-inflammatory cytokine production in LPS-induced AKI.

PGK1 is a key glycolytic enzyme that catalyzes the reversible conversion of 1,3-BPG to 3-PG and contributes to ATP generation during glycolysis [26]. Beyond its canonical metabolic function, accumulating evidence indicates that PGK1 also participates in non-metabolic regulatory processes under stress conditions. PGK1 has been reported to translocate to different cellular compartments and regulate autophagy, mitochondrial function, and redox homeostasis through protein-protein interactions and post-translational modifications [27,28]. Recent studies have also shown that PGK1 expression is upregulated in AKI tissues and may be associated with aggravated renal injury, partly through the regulation of oxidative stress, inflammatory responses, and ferroptosis [29]. In this study, BA directly interacted with PGK1, as supported by CETSA, DARTS, and ITC analyses, with a *K_D_* value of 0.787 μM. Molecular docking further predicted that BA could bind within the active pocket of PGK1. These findings suggest that PGK1 may serve as a molecular target through which BA regulates downstream stress-response pathways.

The Keap1-Nrf2 pathway is a central antioxidant defense mechanism that protects cells against oxidative injury [30]. During AKI progression, excessive ROS production and inflammatory cytokine release can reinforce each other, leading to a damaging cycle of oxidative stress and inflammation. In this study, BA promoted Nrf2 nuclear translocation; restored the expression of Nrf2-regulated antioxidant genes, including *Nqo1*, *Gclc*, and *Gclm*; and reduced ROS accumulation in LPS-stimulated RAW264.7 macrophages. Importantly, the ability of BA to activate the Keap1-Nrf2 pathway and reduce ROS levels was markedly weakened after *Pgk1* knockdown. These findings suggest that BA-mediated antioxidant effects depend, at least in part, on PGK1-associated activation of the Keap1–Nrf2 pathway.

In addition to its antioxidant effects, BA also exhibited anti-inflammatory activity in vivo. H&E staining showed that BA reduced tubular injury and inflammatory cell infiltration in the kidney tissues from mice with LPS-induced AKI. IHC and Western blot analyses further showed that BA decreased the expression of pro-inflammatory mediators, including IL-1β, IL-6, and TNF-α. Consistent with these experimental findings, virtual KO analysis of *Pgk1* in LPS-stimulated macrophages revealed significant changes in immune- and inflammation-related genes. Among these genes, *Il1rn*, *Hmox1*, *Ccl2*, and *Ccl7* showed prominent perturbation effects, suggesting that PGK1 may participate in macrophage inflammatory regulation. GO BP enrichment analysis further supported the involvement of PGK1 in immune system processes, defense responses, and responses to biotic stimuli. These results provide additional evidence that PGK1 is functionally associated with macrophage inflammatory responses under LPS stimulation.

Taken together, this study provides evidence that BA alleviates LPS-induced AKI by reducing oxidative stress and inflammation through PGK1-associated activation of the Keap1-Nrf2 pathway. These findings clarify the pharmacological basis of *O. himalaicus* in kidney-related disorders and suggest that BA may serve as a potential lead compound for the development of mechanism-based nephroprotective agents.

Our study has some limitations. First, the in vitro mechanism and functional validation in this study were mainly carried out using RAW264.7 macrophages. Although macrophages are the core drivers of the LPS-induced AKI inflammatory cascade and cytokine storm, we have not yet directly verified the PGK1-Keap1-Nrf2 pathway in renal parenchymal cells, such as renal tubular epithelial cells. Future studies should further evaluate the direct role of this pathway in cell injury and kidney repair in renal parenchymal cell lines. Second, this study lacks in vivo target knockdown validation. The binding, interaction, and knockdown functional validation between BA and the target PGK1 have only been completed at the cellular and purified protein levels. Although we observed activation of this mechanism axis in overall kidney tissue in mice, due to experimental duration and conditions, we have not yet been able to use macrophage-specific or renal tubular-specific Pgk1 knockout mice to prove that BA’s in vivo efficacy completely depends on direct binding to PGK1. This represents an important direction for future investigation. Finally, future investigations comparing BA with structurally related analogs, together with medicinal chemistry optimization, will be essential for identifying the key structural features responsible for PGK1 binding and the associated biological activity.

## 5. Conclusions

This study demonstrated that BA, a major constituent of *O. himalaicus*, attenuated LPS-induced AKI by reducing oxidative stress and inflammatory responses. Mechanistically, BA directly interacted with PGK1 and promoted activation of the Keap1-Nrf2 pathway, leading to enhanced antioxidant defense and reduced pro-inflammatory cytokine production. These findings provide mechanistic evidence for the renoprotective activity of BA and help clarify the pharmacological basis of *O. himalaicus* in kidney-related disorders. BA may therefore serve as a potential lead compound for the development of mechanism-based nephroprotective agents.

## Data Availability

The data presented in this study are available in this article and its supplementary materials. The RNA-seq analysis presented in the study are openly available in zenodo at https://sandbox.zenodo.org/records/514681 (accessed on 17 June 2026).

## Supplementary Materials

The following supporting information can be downloaded at https://www.mdpi.com/article/10.3390/antiox15070900/s1. Figure S1. Structure of boeravinone A was elucidated by 1H-NMR and 13C-NMR. (A) 1H-NMR spectrum (400 MHz) of boeravinone A in DMSO-d6; (B) 13C-NMR spectrum (100 MHz) of boeravinone A in DMSO-d6; Table S1. Primer sequence (5′-3′) information; Table S2. Primer sequence (5′-3′) of shRNA.
