# Supplementary material for: Boeravinone A Alleviates Oxidative Stress and Inflammation in LPS-Induced Acute Kidney Injury by Targeting PGK1
Source: Antioxidants (Basel). 2026 Jul 20;15(7):900. doi: 10.3390/antiox15070900 (PMC13405194; doi:10.3390/antiox15070900)
Supplement: Supplementary file 1 [file antioxidants-15-00900-s001.zip › antioxidants-4415716-supplementary.pdf]

Boeravinone A alleviates oxidative stress and inflammation in LPS-induced acute kidney injury by targeting PGK1

Supporting Figures

A

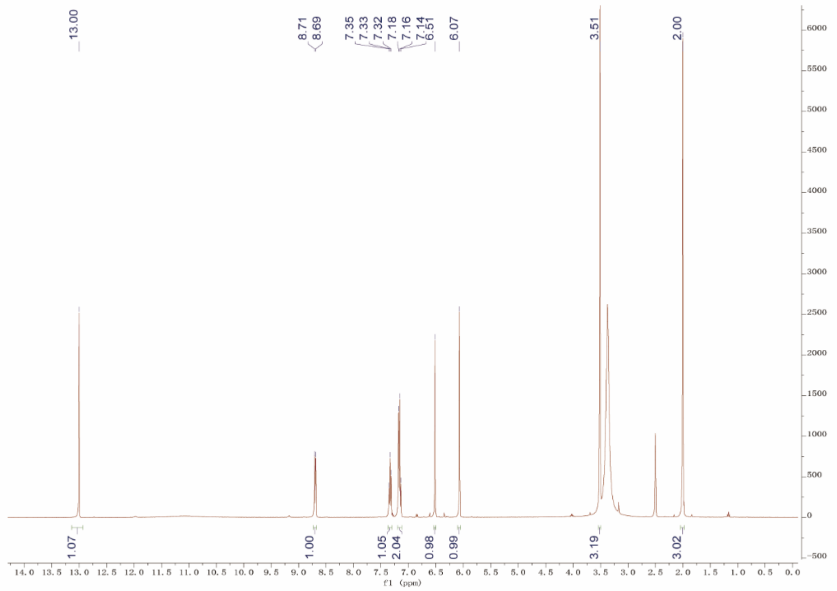

B

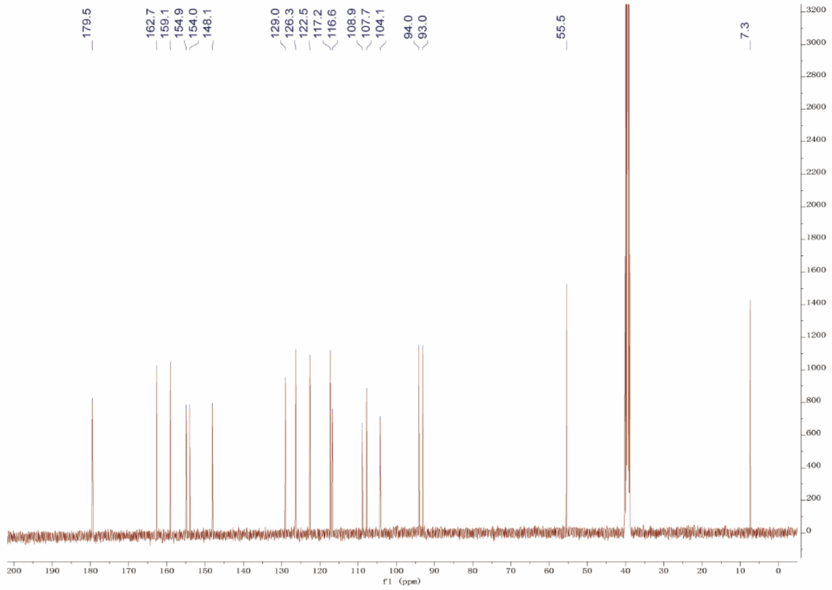

Figure S1. Structure of boeravinone A was elucidated by 1H-NMR and 13C-NMR. (A) 1H-NMR spectrum (400 MHz) of boeravinone A in DMSO-d6; (B) 13C-NMR spectrum (100 MHz) of boeravinone A in DMSO-d6

2. Supporting Tables

Table S1. Primer sequence (5′-3′) information

Primer	Sequence (5′-3′)	Blast accession with 100% query cover	Description
Nfe2l2-F	GCATGATGGAC TTGGAGTTGC	NM_001399226.1	*Mus musculus* nuclear factor, erythroid derived 2, like 2 (Nfe2l2), transcript variant 2, mRNA
		NM_010902.5	*Mus musculus* nuclear factor, erythroid derived 2, like 2 (Nfe2l2), transcript variant 1, mRNA
Nfe2l2-R	AAACTTGTACC GCCTCGTCT	NM_001399226.1	*Mus musculus* nuclear factor, erythroid derived 2, like 2 (Nfe2l2), transcript variant 2, mRNA
Nqo1-F	AGATTAGGAGC CTCAGGGCA	XM_036153810.1	*Mus musculus* NAD(P)H dehydrogenase, quinone 1 (Nqo1), transcript variant X1, mRNA
		NM_008706.5	*Mus musculus* NAD(P)H dehydrogenase, quinone 1 (Nqo1), mRNA
Nqo1-R	TAGCCCGAAG AAAATGGCGA	XM_036153810.1	*Mus musculus* NAD(P)H dehydrogenase, quinone 1 (Nqo1), transcript variant X1, mRNA
		NM_008706.5	*Mus musculus* NAD(P)H dehydrogenase, quinone 1 (Nqo1), mRNA
Gclm-F	GGCGGCTTGAT GCTTTAACA	NM_008129.4	*Mus musculus* glutamate-cysteine ligase, modifier subunit (Gclm), mRNA
Gclm-R	CCAACTGAGCA GCAACACAC	NM_008129.4	*Mus musculus* glutamate-cysteine ligase, modifier subunit (Gclm), mRNA
Actb-F	GATCAGCAAG CAGGAGTACG A	NM_007393.5	*Mus musculus* actin, beta (Actb), mRNA
Actb-R	GGGTGTAAAAC GCAGCTCA	NM_007393.5	*Mus musculus actin*, beta (Actb), mRNA
Keap1-F	GATATGAGCCA GAGCGGGAC	NM_001110307.1	*Mus musculus* kelch-like ECH-associated protein 1 (Keap1), transcript variant 4, mRNA
		NM_001110305.1	*Mus musculus* kelch-like ECH-associated protein 1 (Keap1), transcript variant 2, mRNA
		NM_016679.4	*Mus musculus* kelch-like ECH-associated protein 1 (Keap1), transcript variant 1, mRNA
		NM_001110306.1	*Mus musculus* kelch-like ECH-associated protein 1 (Keap1), transcript variant 3, mRNA
Keap1-R	CATACAGCAA GCGGTTGAGC	NM_001110307.1	*Mus musculus* kelch-like ECH-associated protein 1 (Keap1), transcript variant 4, mRNA
		NM_001110305.1	*Mus musculus* kelch-like ECH-associated protein 1 (Keap1), transcript variant 2, mRNA

		NM_016679.4	*Mus musculus* kelch-like ECH-associated protein 1 (Keap1), transcript variant 1, mRNA
		NM_001110306.1	*Mus musculus* kelch-like ECH-associated protein 1 (Keap1), transcript variant 3, mRNA
Gclc-F	TGGCCACTATC TGCCCAATT	NM_010295.2	*Mus musculus* glutamate-cysteine ligase, catalytic subunit (Gclc), transcript variant 1, mRNA
		XM_006510812.2	*Mus musculus* glutamate-cysteine ligase, catalytic subunit (Gclc), transcript variant X1, mRNA
Gclc-R	GTCTGACACGT AGCCTCGGTAA	NM_010295.2	*Mus musculus* glutamate-cysteine ligase, catalytic subunit (Gclc), transcript variant 1, mRNA
		XM_006510812.2	*Mus musculus* glutamate-cysteine ligase, catalytic subunit (Gclc), transcript variant X1, mRNA

Table. S2. Primer sequence (5′-3′) of shRNA

Forward oligo	CCGGGCTATCTTGGGAGGCGCTAAACTCGAGTTTAGCGCCTCCCA AGATAGCTTTTTG
Reverse oligo	AATTCAAAAAGCTATCTTGGGAGGCGCTAAACTCGAGTTTAGCG CCTCCCAAGATAGC
